# Dynamic effects of cholinergic blockade upon cerebral blood flow autoregulation in healthy adults

**DOI:** 10.3389/fphys.2022.1015544

**Published:** 2022-11-02

**Authors:** Vasilis Z. Marmarelis, Dae C. Shin, Jason W. Hamner, Can Ozan Tan

**Affiliations:** ^1^ Biomedical Engineering, University of Southern CA, Los Angeles, MA, United States; ^2^ Cardiovascular Research Laboratory, Spaulding Rehabilitation Hospital, Boston, MA, United States; ^3^ Electrical Engineering Math and Computer Science, University of Twente, Enschede, Netherlands

**Keywords:** cerebral blood flow regulation, cerebral autoregulation, dynamic vasomotor reactivity, autonomic control, cholinergic mechanism

## Abstract

**Background:** Cerebral flow autoregulation (CFA) is a homeostatic mechanism critical for survival. The autonomic nervous system (ANS) plays a key role in maintaining proper CFA function. More quantitative studies of how the ANS influences CFA are desirable.

**Objective:** To discover and quantify the dynamic effects of cholinergic blockade upon CFA in response to changes of arterial blood pressure and blood CO2 tension in healthy adults.

**Methods:** We analyzed time-series data of spontaneous beat-to-beat mean arterial blood pressure (ABP) and cerebral blood flow velocity in the middle cerebral arteries (CFV), as well as breath-to-breath end-tidal CO2 (CO2), collected in 9 adults before and after cholinergic blockade, in order to obtain subject-specific predictive input-output models of the dynamic effects of changes in ABP and CO2 (inputs) upon CFV (output). These models are defined in convolutional form using “kernel” functions (or, equivalently, Transfer Functions in the frequency domain) that are estimated *via* the robust method of Laguerre expansions.

**Results:** Cholinergic blockade caused statistically significant changes in the obtained kernel estimates (and the corresponding Transfer Functions) that define the linear dynamics of the ABP-to-CFV and CO2-to-CFV causal relations. The kernel changes due to cholinergic blockade reflect the effects of the cholinergic mechanism and exhibited, in the frequency domain, resonant peaks at 0.22 Hz and 0.06 Hz for the ABP-to-CFV and CO2-to-CFV dynamics, respectively.

**Conclusion:** Quantitative estimates of the dynamics of the cholinergic component in CFA are found as average changes of the ABP-to-CFV and CO2-to-CFV kernels, and corresponding Transfer Functions, before and after cholinergic blockade.

## Introduction

The role of the autonomic nervous system in the autoregulation of cerebral blood flow, during changes of blood pressure and metabolic demands, has been a matter of long-standing scientific interest (Willie et al., 2014; Tan and Taylor, 2014; Hamner et al., 2012; Hamner et al., 2010; Zhang et al., 2002; Azevedo et al., 2011; ter Laan et al., 2013; Edvinsson, 1975; Hamner and Tan, 2014; Hamner et al., 2010; Sagawa and Guyton, 1961; Seifert et al., 2010; Strandgaard and Sigurdsson, 2008; van Lieshout and Secher, 2008). The effects of autonomic control on cerebral blood flow dynamics are overlaid on the contemporaneous effects of myogenic, metabolic and endocrine mechanisms, in addition to the complicated effects of neurovascular coupling. Previous studies have established causative relations between cholinergic activity and cerebral blood flow and/or arterial blood pressure (Edvinsson, 1975; Zhang et al., 2002; Seifert et al., 2010; Hamner et al., 2012; Hamner and Tan, 2014; Tan and Taylor, 2014; Willie et al., 2014), although they have been non-predictive or used static relations (linear or nonlinear). Quantitative and predictive descriptions of the dynamic characteristics of these causative relations have been limited (Zhang et al., 2002). This provides the motivation for the present work that is focused on estimating the linear dynamics of cholinergic control on cerebral blood flow.

It has long been known that the cerebral vasculature is innervated with both adrenergic and cholinergic fibers (Edvinsson, 1975), but their distinct and separate (or synergistic) role in cerebral flow autoregulation has been hotly debated (Strandgaard and Sigurdsson, 2008; van Lieshout and Secher, 2008). In the 1960s Guyton and others isolated the cerebral circulation of one dog, eliminating all autonomic input, by connecting it to the peripheral circulation of a different dog whose carotid sinus nerves had been cut (Sagawa and Guyton, 1961). The recipient dog passively responded to pressure changes, showing no signs of cerebral autoregulation to blood pressure changes. In humans, Zhang et al, (2002) first provided evidence of autonomic involvement in cerebral autoregulation by looking at transfer function relations with and without full ganglionic blockade. Subsequent work has teased apart the different roles of neurogenic mechanisms (Hamner and Tan, 2014) and observed distinct, as well as vital, roles for both adrenergic (Hamner et al., 2010) and cholinergic (Seifert et al., 2010; Hamner et al., 2012) contributions to cerebral flow autoregulation.

The present work seeks to obtain quantitative measures of the dynamic effects of cholinergic activity upon cerebral flow autoregulation (CFA) by estimating data-based linear predictive models of the dynamic (causal) relation between beat-to-beat mean arterial blood pressure (ABP) or end-tidal CO2, viewed as the putative inputs, and cerebral blood flow velocity (CFV) at the right middle cerebral artery, viewed as the putative output—before and after cholinergic blockade. These models contain two terms that represent the dynamic relations for ABP-to-CFV and CO2-to-CFV in the form of convolutions of each input with the respective “kernel” function. The Fourier Transform of each kernel corresponds to the Transfer Function for the respective input-output relation. The difference between the Transfer Functions of each subject, before and after blockade, is deemed to represent a quantification of the dynamic effects of cholinergic activity upon the respective branch of CFA under resting conditions.

In our previous studies, we have shown that these two-input/one-output models can be obtained at resting-state from spontaneous activity time-series data of ABP, CO2 and CFV (Marmarelis et al., 2017; Marmarelis et al., 2020a; Marmarelis et al., 2020b). To obtain robust estimates of such dynamic models, we employed the kernel-based modeling methodology using Laguerre expansions (Marmarelis, 1993; Marmarelis, 2004) that is outlined in the following section. The reported results indicate impaired CO2 dynamic vasomotor reactivity (pertaining to the CO2-to-CFV relation) in patients with mild cognitive impairment or essential hypertension (Marmarelis et al., 2017; Marmarelis et al., 2020a; Marmarelis et al., 2020b). Impaired dynamic cerebral autoregulation in response to ABP changes (pertaining to the ABP-to-CFV relation) was observed in the patients with essential hypertension (Marmarelis et al., 2020a) but not in MCI patients (Marmarelis et al., 2017; Marmarelis et al., 2020b). Laguerre expansions for the study of the ABP-to-CFV dynamics were first used in (Panerai et al., 1999). In seeking the physiological mechanisms that partake in these impairments, we performed the analysis of Principal Dynamic Modes (PDMs) that decomposes the system kernels into an orthogonal representation of steepest convergence (Marmarelis, 2004). This study seeks to reveal which PDMs are affected mostly by cholinergic blockade (thus containing cholinergic components) and to begin the process of physiological interpretation of the PDMs of CFA.

We note that the term “cerebral autoregulation” (CA or CAR) has been used by the peer community to describe the dynamic relation between ABP and CFV in the extensive literature on this matter [e.g. (Aaslid et al., 1989; Paulson et al., 1990; Tiecks et al., 1995; Panerai, 1998; Zhang et al., 1998; Panerai et al., 1999; van Beek et al., 2008; Mitsis et al., 2009; Porta et al., 2022)]. The term “cerebral flow autoregulation” (CFA) is used in this paper to draw the distinction with those previous studies with regard to the use of a second input (CO2) accounting for the contemporaneous effects on CFV of spontaneous changes in blood CO2 tension, as well as to make the object of autoregulation explicit.

## Materials and methods

### Modeling methodology

The quantification of physiological function that is described by a linear two-input/one-output dynamic model can be achieved through estimation of the two “kernels” (or “Impulse Response Functions”) that define the relation between each input and the output according to the equation:

$$y\left(n\right)=k_{0}+\Sigma_{m}\;k_{p}\left(m\right)p\left(n-m\right)+\Sigma_{m}\;k_{x}\left(m\right)x\left(n-m\right)+\varepsilon \left(n\right)$$ (1)

where *y(n)* denotes the output signal (CFV), and *p(n)* and *x(n)* denote the ABP and CO2 input signals, respectively. The prediction error (residual term) is denoted as *ε(n)*. The kernels *k*_*p*_ and *k*_*x*_ in the model (also termed the “Impulse Response Functions”) describe fully the dynamic characteristics of the ABP-to-CFV and CO2-to-CFV relations, respectively. The kernels are functions of the “lag” variable “*m*”, which denotes the time preceding the present-time at the output for the corresponding kernel values, *k*_*p*_ *(m)* and *k*_*x*_ *(m)*, to influence as weights the generated present value of the output, according to the weighted sum indicated in the respective convolutions of Eq. 1. For instance, if a system simply delays by *D* time-units the input to generate the output (without any alterations in waveform), then its kernel only has a discrete unity impulse at lag *D*. Of course, physiological systems generally alter the input waveform to generate the output waveform (dynamic transformation) and, therefore, have kernels with a continuum of values (between 0 lag and the “effective memory” of the system) defining the relative weight by which an instantaneous change in the input will affect the output. For this reason, the kernel is also called the “Impulse Response Function”. Thus, the kernel waveform depicts the relative size of the effects caused by the past (and present) input values upon the output present value. The “effective memory” of a physiological system is the minimum time required for the effect of an impulsive input upon the output to become negligible. Upon estimation of the kernels from input-output data, the linear model of Eq. 1 allows prediction of the output *y(n)* for *any* given input waveforms *p(n)* and *x(n)*. This model form is generally valid for all linear and stationary (or time-invariant) systems. However, the presented methodological approach can be extended to nonlinear and nonstationary systems, provided certain practical requirements are met to achieve reliable estimates (Marmarelis, 1993; Mitsis et al., 2002; Marmarelis, 2004; Claassen et al., 2016).

To obtain robust estimates of the kernels of this model, we employ the method of Laguerre expansion of kernels that has been shown to yield predictive models (linear or nonlinear) even for noisy and relatively short datasets (Marmarelis, 1993; Marmarelis, 2004). This methodology is briefly outlined in the Supplementary Appendix SA, where it is shown that the kernels can be estimated through linear regression of a modified model equation using Laguerre expansions. The Transfer Function estimates are obtained *via* Discrete Fourier Transform of the respective kernel estimates.

To check the statistical significance of the obtained kernel estimates, we test the statistical Null Hypothesis of “no causal dynamic input-output relation contained within the time-series data” by estimating the kernels using the given input time-series data and *randomly shuffled* output time-series data for 1000 independent trials in order to obtain an ensemble of kernel estimates that correspond to the Null Hypothesis. This ensemble is used to estimate the standard deviation (SD) bounds for all lags (the mean is zero for all lags under the Null Hypothesis). Then, we use the values of the actual kernel estimates to run a two-sided t-test against the Null Hypothesis over all lags from 0 to the effective memory of the kernel. If the actual kernel estimate rejects the Null Hypothesis at the 95% confidence level *at any lag*, then the Null Hypothesis is rejected for the entire kernel estimate and the latter is considered to capture a statistically significant causal relation between the time-series data of the input and the output (at the 95% confidence level). This statistical procedure is applied to all kernel estimates obtained from our input-output data to establish their statistical significance.

### Principal dynamic mode analysis

The PDMs represent an orthogonal basis for kernel decomposition into components that contribute to the kernel estimates of a cohort in uncorrelated manner and a ranked descending order of steepest convergence—i.e. they can be viewed as a linear transformation (rotation) of the Laguerre function basis that achieves the best subspace approximation of the cohort kernels for a given subspace dimension and having diagonal covariance matrix (Marmarelis, 2004). Thus, the PDMs can be derived through Singular Value Decomposition of a rectangular matrix containing as column vectors all kernel estimates of the cohort (see Supplementary Appendix SA). Aside of their mathematical and operational importance, the PDMs raise the intriguing question of whether they possibly correspond to specific physiological mechanisms. This question can be answered only empirically (i.e. from data of specific systems for which sufficient knowledge exists about their physiological mechanisms). The answer to this important question is expected to vary from application to application. The present paper offers an opportunity to study the effect of cholinergic blockade upon the PDMs of the CFA system.

### Data collection

The analyzed time-series data were collected for a previously published study (Hamner et al., 2012) during 5 min supine resting position. Subjects were 9 healthy adults, who participated voluntarily in this study and signed the Informed Consent Form that had been approved by the Institutional Review Board of the Spaulding Rehabilitation Hospital. The study conformed to the *Declaration of Helsinki*. The volunteers were 9 healthy adults (4 female and 5 male) with ages ranging from 21 to 30 (27.1 ± 0.77) years and BMI ranging from 20 to 30 (24.4 ± 0.97). The participants were normotensive non-smokers without cardiovascular or neurological disorders and were not taking cardioactive medications. The participants also refrained from alcohol, caffeine and rigorous exercise at least 24 h prior to the study. Data were collected before cholinergic blockade and 30 min after cholinergic blockade. The gender, age, and time-average mean (SD) of the ABP, CO2, heart-rate (HR) and CFV data of the 9 participants are given in Table 1.

**TABLE 1:** Gender, age and time-average mean (SD) of ABP, CO2, CFV & HR data of 9 subjects.

Subjects:9 healthy adults (4 Female 5 Male)	Age	ABP	CO2	CFV	HR
Before blockade (baseline)	27.10 (0.77)	83.82 (6.09)	38.34 (4.83)	64.48 (7.51)	65.61 (8.17)
After cholinergic blockade		96.43 (10.58)	36.54 (6.64)	67.14 (7.92)	101.90 (7.41)
p-value for paired t-test	NA	0.0179	0.0692	0.0129	2.804×10–8

The data collection protocol consisted of 5 min of resting measurements. For each subject, a 20-gauge catheter was inserted into an antecubital vein for drug infusion. Subsequently, each subject was instrumented for electrocardiogram lead II (Dash 2000; General Electric), photoplethysmography for arterial blood pressure (Portapres, Finapres Medical Systems) and oscillometric brachial pressures (DASH 2000; General Electric) taken as a check for photoplethysmographic finger pressures. Each subject was also instrumented for measurement of blood flow velocity in the middle cerebral artery *via* transcranial Doppler (2 and 4 MHz probes; Multidop T2, DWL). A custom-made probe fixation device held the Doppler probe in place. Expired CO2 was monitored *via* an infrared carbon dioxide analyzer (CO2 Analyzer Model 17,515, Vacumed) connected to a nasal cannula. All signals were digitized and stored at 1000 Hz (PowerLab, AD Instruments).

### Data preprocessing

**FIGURE 1:**
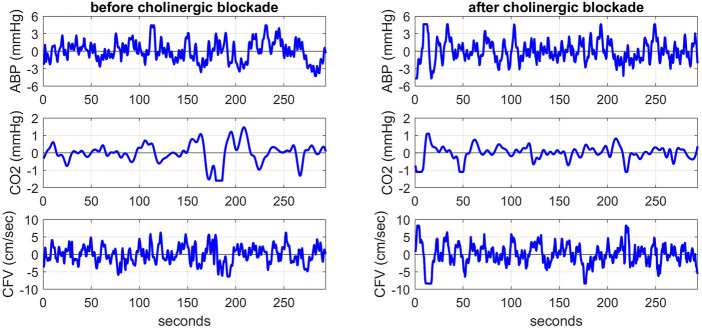
Illustrative pre-processed time-series data of ABP (top panels), CO2 (middle panels) and CFV (bottom panels) at the resting state for one subject before (left) and after (right) cholinergic blockade. The units are: mmHg for ABP and CO2, and cm/sec for CFV.

Highly sampled time-series data of ABP and CFV were collected at 50 Hz and down-sampled to beat-to-beat time-series data using averages over the respective R-R intervals (i.e. the sum of the highly sampled values over each R-R interval divided by the R-R interval length). These beat-to-beat averages of ABP and CFV were placed at the mid-point of each R-R interval (unevenly spaced). The breath-to-breath end-tidal CO2 measurements were placed at the mid-point of each breath (unevenly spaced) and shifted to the left by 3 s to account for the tube delay of the capnograph that was measured separately for the standard tube length. After removing occasional outliers due to measurement artifacts through application of hard-clipping at ±2.5 standard deviations about the time-average values, we re-sampled the data every 0.5 s using cubic-spline interpolation to obtain even sampling rate (necessary for subsequent processing). The resulting evenly-sampled time-series data were subsequently high-pass filtered (*via* subtraction of a 2-min moving-average using a Hanning window) to remove the time-average (DC) value and very low frequency content below 0.01 Hz. Figure 1 shows illustrative evenly-sampled pre-processed time-series data resulting from the described procedure for one of the subjects before (left) and after (right) cholinergic blockade. The inclusion of *“(t)”* next to each variable name is intended to distinguish these time-series data from the original raw measurements.

## Results

**FIGURE 2:**
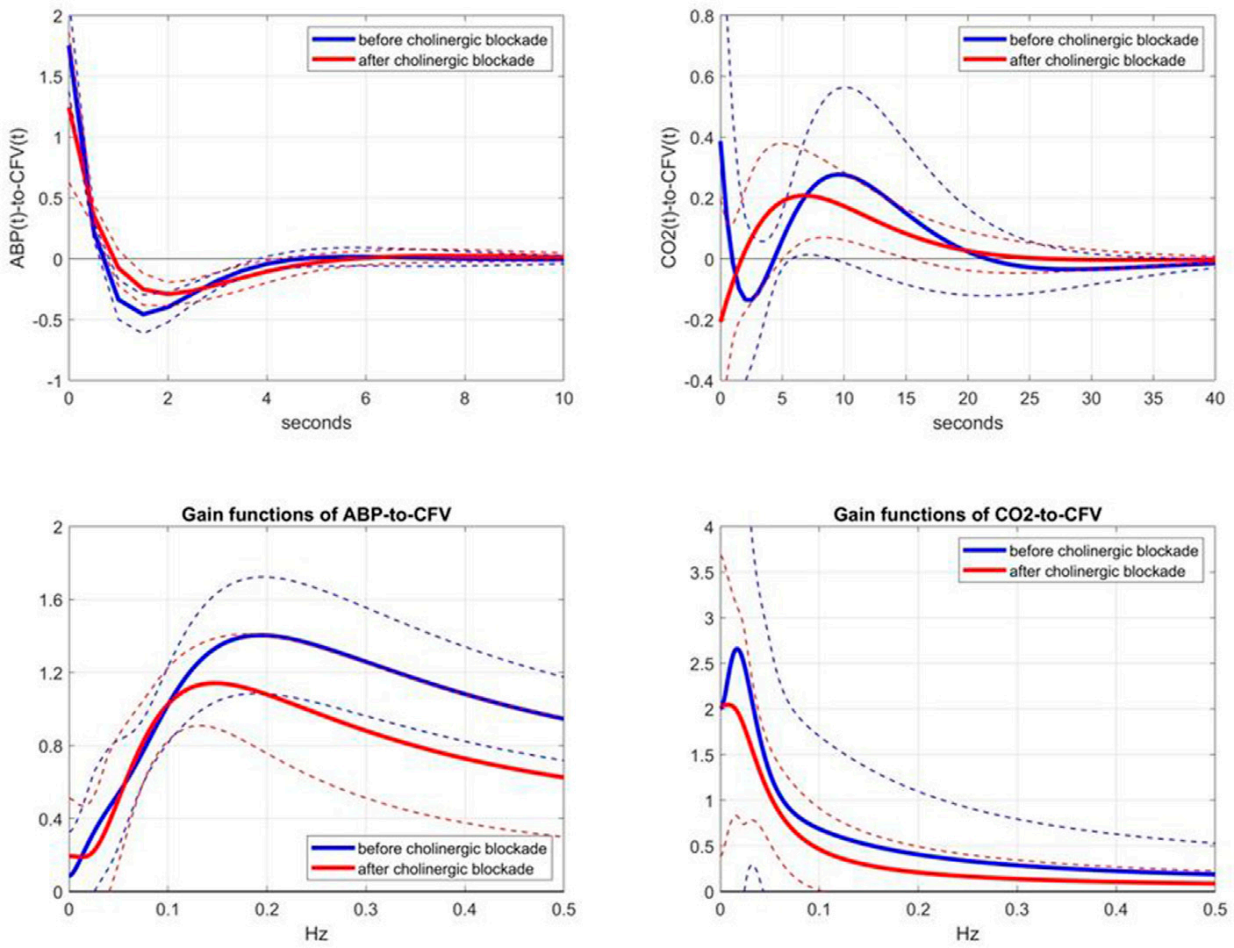
Top panels: Average kernel estimates (±1 SD bounds in dotted lines) for the ABP-to-CFV relation (left) and CO2-to-CFV relation (right) over 9 subjects before (blue line) and after (red line) cholinergic blockade. Bottom panels: Average Gain Functions (±1 SD bounds in dotted lines) for the ABP-to-CFV relation (left) and CO2-to-CFV relation (right) over 9 subjects before (blue line) and after (red line) cholinergic blockade, which are computed *via* Discrete Fourier Transform of the respective kernels.

Following the aforementioned modeling methodology, we obtained the kernel estimates for the ABP-to-CFV and CO2-to-CFV dynamic relations in the 9 subjects before and after cholinergic blockade, which are shown in Figure 2 (top panels), along with the respective Gain Functions (i.e. magnitudes of their Discrete Fourier Transforms) (bottom panels). Some kernel differences are seen after blockade that are discussed later. For a measure of prediction accuracy provided by these kernel estimates, we use the Normalized Mean-Square Error (NMSE), which is the mean-square value of the prediction error (residuals) divided by the mean-square value of the demeaned output data. The NMSE values of the output predictions provided by the two sets of kernels in this study have mean (SD) values of: 41.86 (12.89) before blockade vs 57.07 (14.62) after blockade, resulting in a *p*-value of 0.032. This result indicates that the predictability of CFV changes from ABP and CO2 changes becomes significantly reduced after cholinergic blockade.

We consider the observed changes of the kernel estimates after cholinergic blockade to represent the dynamic effects of the cholinergic mechanism in the two CFA pathways: ABP-to-CFV and CO2-to-CFV. Figure 2 shows that the effect of cholinergic blockade on the average ABP-to-CFV kernel is easier to describe in the frequency domain (Gain Function) as a downshift of the resonant-peak frequency from about 0.20 Hz to about 0.14 Hz and a reduction of the maximum Gain by about 25%. For the average CO2-to-CFV kernel, the effect of cholinergic blockade is mainly seen as a reduction of Gain across all frequencies and the downshift of the resonant-peak around 0.02 Hz before blockade to about 0.01 Hz after blockade.

**FIGURE 3:**
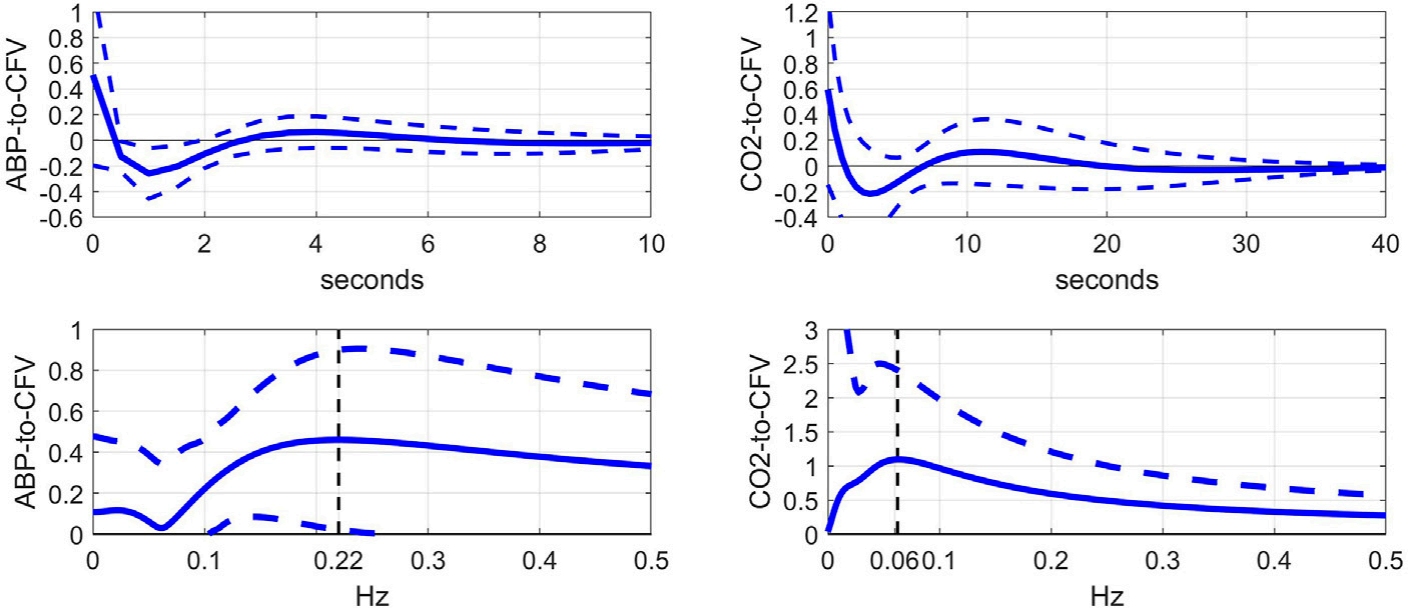
Top: Average difference of kernel estimates (±1 SD bounds in dotted lines) before minus after cholinergic blockade for the ABP-to-CFV (left) and CO2-to-CFV (right) relations over 9 subjects. These kernel differences may describe the dynamics of the cholinergic mechanism if its effects are additive. Bottom: DFT magnitudes (±1 SD bounds in dotted lines) of the average kernel differences for the ABP-to-CFV (left) and CO2-to-CFV (right) relations. They indicate that the cholinergic mechanism may exhibit a resonant peak around 0.22 Hz for the ABP-to-CFV relation (the neighborhood of average breathing frequency and vagal drive) and around 0.06 Hz for the CO2-to-CFV relation. The latter frequency is also the location of the minimum value in the ABP-to-CFV DFT magnitude and remains open to interpretation (see Discussion).

This kernel comparison (before vs after blockade) invites the examination of the kernel differences, which are shown in Figure 3 (top panels), along with the respective DFT magnitudes (bottom panels). In the ABP-to-CFV pathway (left panels), the kernel difference suggests that the cholinergic mechanism may exhibit a resonance around 0.22 Hz, which is consistent with the downshift of the resonant-peak seen in Figure 2 and the widely held view that cholinergic blockade should have a strong effect around the average breathing frequency. In the CO2-to-CFV pathway (right panels), the cholinergic mechanism appears to exhibit a resonance around 0.06 Hz, which is the same frequency where a minimum appears in the ABP-to-CFV pathway (see Discussion).

**FIGURE 4:**
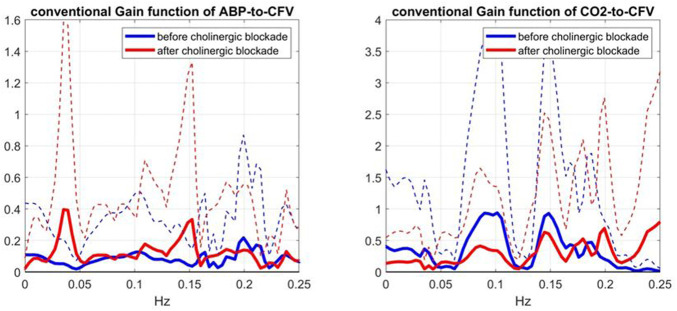
Average Gain Functions (SD bounds in dotted lines) obtained *via* the conventional Transfer Function analysis method (Claassen et al., 2016) for the ABP-to-CFV (left) and CO2-to-CFV (right) relations over 9 subjects before (blue line) and after (red line) cholinergic blockade.

The comparison of the obtained Gain Functions *via* the Laguerre expansion technique before and after blockade (shown in the bottom panels of Figure 2) invites the comparison with the Gain Functions estimated *via* the conventional Transfer Function analysis (Claassen et al., 2016), which are shown in Figure 4. The observed notable differences between these two types of Gain Function estimates are discussed further in Discussion.

**FIGURE 5:**
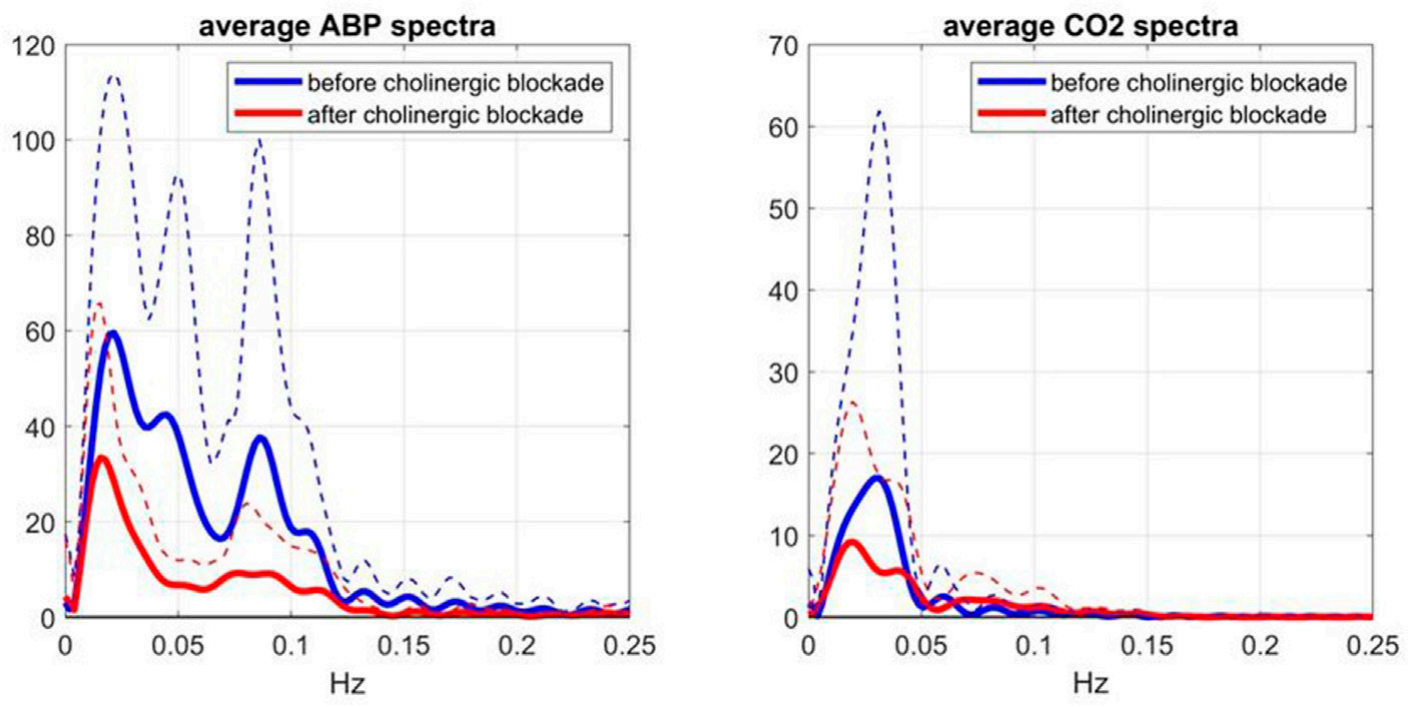
Average Spectra (and SD bounds in dotted lines) of the ABP input (left) and CO2 input (right) over 9 subjects before (blue line) and after (red line) cholinergic blockade, which are computed *via* the conventional method (Claassen et al., 2016).

To evaluate further these conventional Gain Function estimates, it is informative to examine the average input spectra (shown in Figure 5)—since the input spectrum (for ABP or CO2) divides the respective cross-spectrum to yield the conventional Gain Function estimate. Due to this division, frequency ranges with very small input spectral values are expected to yield unreliable conventional Gain Function estimates in the presence of output noise. It is seen that the average ABP input spectrum has significant values only up to 0.12 Hz -- thus calling into question the reliability of the conventional Gain Function peak around 0.15 Hz (after blockade) in the left panel of Figure 4. The average CO2 input spectrum has significant values only up to about 0.05 Hz -- thus calling into question the reliability of the conventional Gain Function peaks around 0.10 Hz and 0.15 Hz before and after blockade (see right panel of Figure 4). We note that the Gain Function estimates obtained *via* the Laguerre expansion technique do not rely on division by the input spectra and have taken into account the contemporaneous effects of the two inputs.

**FIGURE 6:**
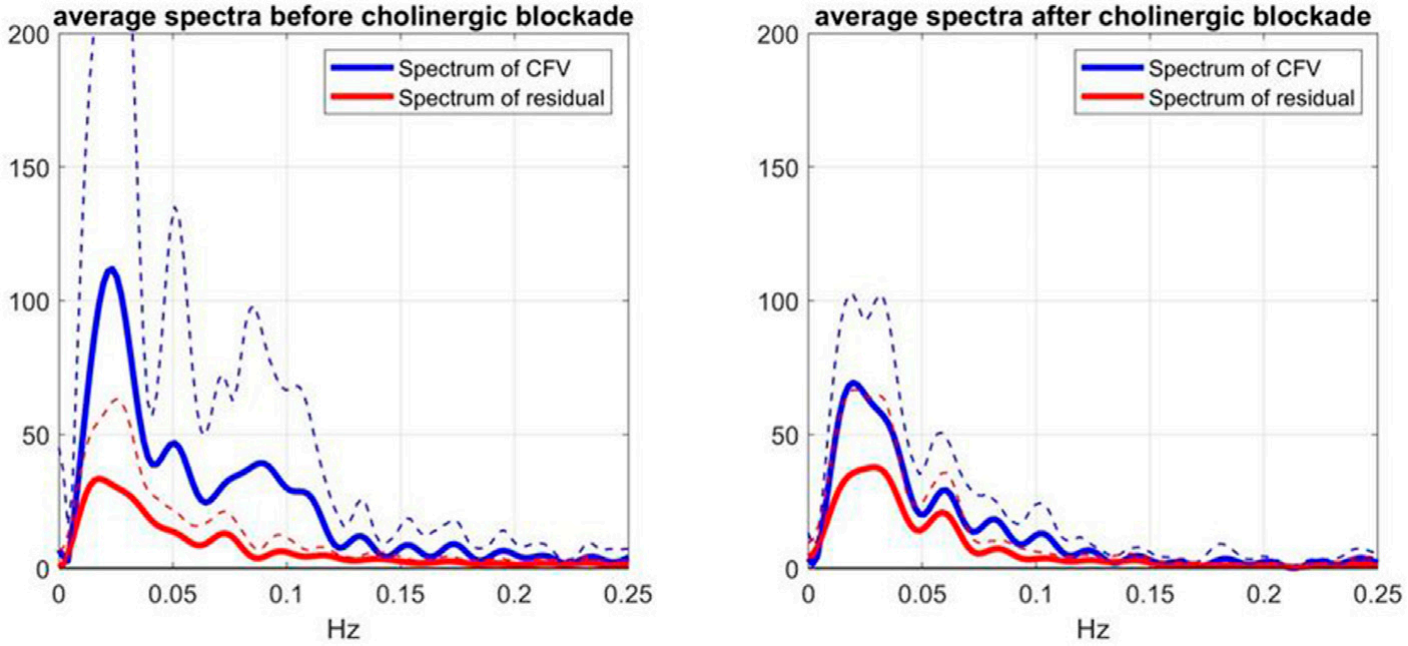
Average Spectra (and SD bounds in dotted lines) of CFV output (blue line) and model-prediction residuals (red line) before blockade (left) and after blockade (right) over 9 subjects.

The effects of cholinergic blockade on the spectral characteristics of the model-prediction residuals are illustrated in Figure 6, where the average spectra and SD boumds of the residuals (red line) and of the CFV output (blue line) are shown before (left panel) and after (right panel) cholinergic blockade—indicating greater variability of CFV before cholinergic blockade (attested of the larger integrated value) and a strong spectral peak of the residuals in the frequency range 0.02–0.03 Hz (i.e. non-white residuals). The latter resonance is also seen in the CFV spectra, along with two weaker spectral peaks in the ranges 0.05–0.06 Hz and 0.08–0.09 Hz.

Notably, the latter CFV spectral peak is far more pronounced before cholinergic blockade.

In order to examine the statistical significance of the effects of cholinergic blockade upon the estimated kernels, we performed the following statistical Hypothesis testing. For each lag of the ABP-to-CFV or CO2-to-CFV kernel (from 0 to the respective memory-extent every 0.5 s), we compute the difference between the estimates before and after blockade for each of the 9 subjects, and subsequently test the mean of this sample of 9 differences against the Null Hypothesis of zero mean (i.e. using a paired two-sided t-test). If the Null Hypothesis gets rejected at 95% confidence level for any lag of the kernel, then the kernel change due to cholinergic blockade (for all lags) is deemed statistically significant. Following this procedure, we found that the cholinergic blockade caused significant change to both kernels.

**FIGURE 7:**
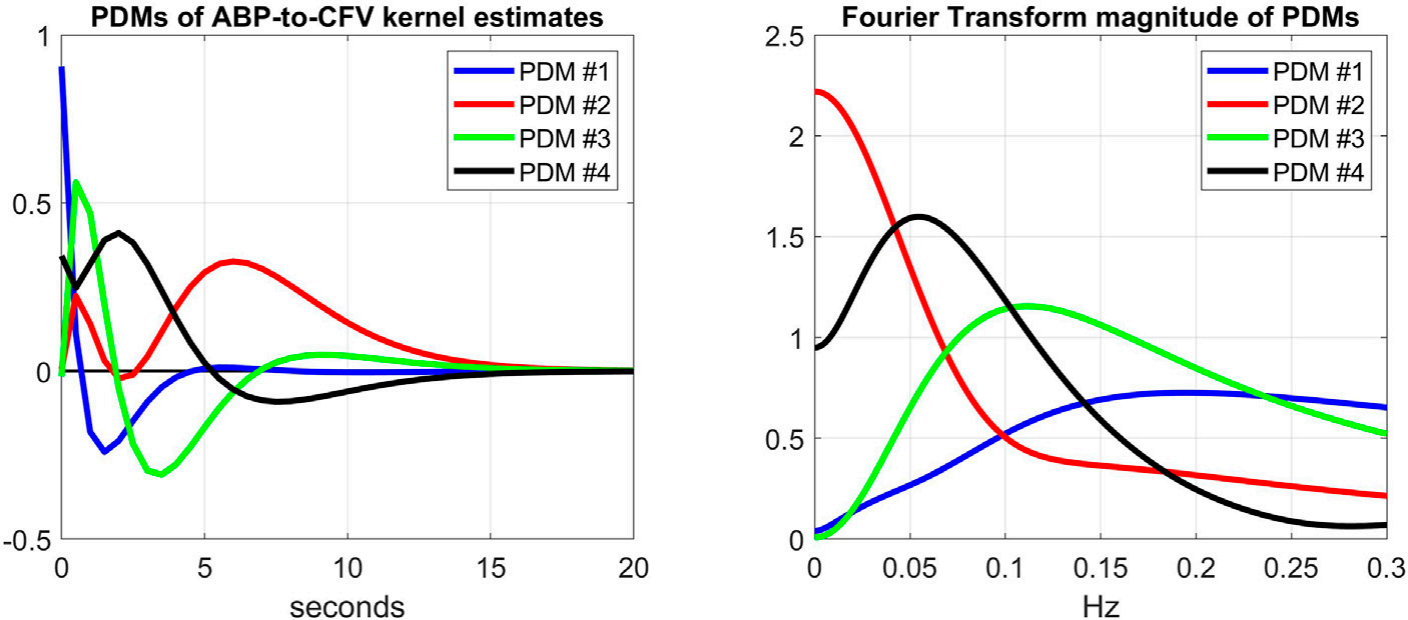
Left: The 4 PDMs of the ABP-to-CFV kernel estimates of the cohort before cholinergic blockade obtained *via* SVD (see Supplementary Appendix SA). The corresponding Singular Values are: 5.92, 0.63, 0.43, 0.41 (signifying the relative importance of each PDM in composing the ABP-to-CFV kernel estimates in the cohort). Right: The Discrete Fourier Transform magnitudes of the 4 PDMs that exhibit distinct high-pass (differentiating) characteristics (1st PDM), low-pass (integrative) characteristics (2nd PDM), and band-pass (resonant) characteristics (3rd and 4th PDMs).

**FIGURE 8:**
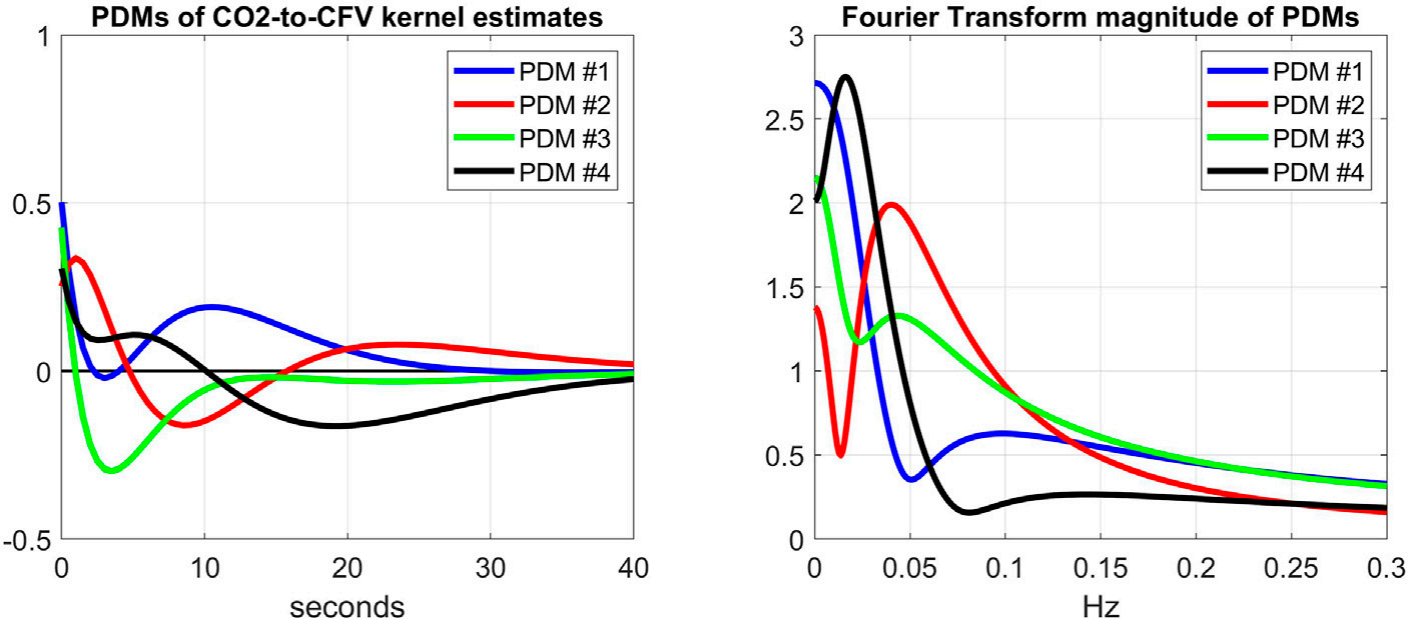
Left: The 4 PDMs of CO2-to-CFV kernel estimates of the cohort before cholinergic blockade obtained *via* SVD (see Supplementary Appendix SA). The corresponding Singular Values are: 5.68, 2.95, 0.74, 0.43; indicating that the 1^st^ and 2^nd^ PDMs are dominant. Right: The Discrete Fourier Transform magnitudes of the 4 PDMs that exhibit a mix of low-pass (integrative) and band-pass (resonant) characteristics, but no high-pass characteristics. The dominant 1^st^ and 2^nd^ PDMs exhibit low-pass and band-pass (resonant peak around 0.04 Hz) characteristics, respectively.

Another way to explore the effects of cholinergic blockade (at rest) upon the dynamics of the CFA system is to examine the kernel decomposition into Principal Dynamic Modes (PDMs) (Marmarelis et al., 2017; Marmarelis et al., 2020a). Following the procedure for PDM analysis that was outlined in Methods, we obtain the PDMs of the ABP-to-CFV and CO2-to-CFV kernels from the cohort of 9 subjects before cholinergic blockade that are shown in Figures 7, 8 (left panels), respectively, along with their frequency-domain counterparts (right panels). We see that the PDMs exhibit distinct high-pass (differentiating) or low-pass (integrative) or band-pass (resonant) characteristics. For the ABP-to-CFV kernel, the Singular Values that correspond to the four PDMs (from the SVD process of PDM analysis) are: 5.92, 0.63, 0.43, 0.41; signifying the relative importance of each of them in composing the ABP-to-CFV kernels for the various subjects. The 1^st^ PDM is dominant in this case, because its corresponding 1^st^ Singular Value (5.92) is an order of magnitude larger than the 2^nd^ Singular Value (0.63). The Singular Values for the CO2-to-CFV kernel PDMs are: 5.68, 2.95, 0.74, 0.43, indicating that the 1^st^ and 2^nd^ PDMs are dominant in this case, as suggested by the respective Singular Values being much larger than the 3^rd^ and 4^th^

When we expand/decompose the kernel estimates of the cohort (before and after blockade) upon the basis of the PDMs, we obtain the expansion coefficients whose mean and SD values are shown in Table 2 for the ABP-to-CFV relation and in Table 3 for the CO2-to-CFV relation. The corresponding *p*-values for paired t-test (comparing the coefficients of each PDM before vs. after blockade) are also shown in these Tables and indicate significant differences in the 1st and 3rd PDM coefficients for the ABP-to-CFV relation and in the 3rd PDM coefficient for the CO2-to-CFV relation.

**TABLE 2:** Mean (SD) values of PDM expansion coefficients of the ABP-to-CFV kernel estimates before and after cholinergic blockade. Δ denotes the mean difference before minus after blockade.

ABP	PDM #1		PDM #2		PDM #3		PDM #4	
	Before	After	Before	After	Before	After	Before	After
µ (ϭ)	1.9324 (0.4362)	1.3695 (0.6009)	−0.0013 (0.2249)	0.0507 (0.1655)	0.0253 (0.1497)	0.3334 (0.2025)	0.0122 (0.1445)	0.0302 (0.2895)
Δ	0.5628 (0.6955)		−0.0520 (0.2825)		−0.3081 (0.3004)		−0.0180 (0.2999)	
P	0.04135		0.59604		0.01520		0.86167	

**TABLE 3:** Mean (SD) values of PDM expansion coefficients of the CO2-to-CFV kernel estimates before and after cholinergic blockade. Δ denotes the mean difference before minus after blockade.

CO2	PDM #1		PDM #2		PDM #3		PDM #4	
	Before	After	Before	After	Before	After	Before	After
µ (ϭ)	1.0295 (1.6891)	0.3906 (0.4813)	−0.5408 (0.8735)	−0.4131 (0.3373)	−0.0110 (0.2634)	−0.6616 (0.7326)	0.0338 (0.1500)	−0.0601 (0.3323)
Δ	0.6389 (1.5157)		−0.1278 (0.9091)		0.6506 (0.7034)		0.0939 (0.3617)	
P	0.24161		0.68433		0.02411		0.45838	

These findings indicate that the projections of the kernel changes due to cholinergic blockade (see Figure 3) upon the PDM bases are significant for two PDMs of the ABP-to-CFV relation, which suggests that a single PDM may not correspond to a distinct physiological mechanism and *vice versa* (see Discussion).

Finally, one may reason that the quantitative effect of the cholinergic blockade upon the PDMs of the CFA dynamics may be better assessed on the basis of the contributions of the various PDMs to the CFV model-predicted output (i.e. the convolution of each PDM with its respective input) rather than the expansion coefficients of the PDMs. The difference is that the PDM contributions take into account the spectral characteristics of the specific input data that were used for kernel (and PDM) estimation. This can make considerable difference in the result, since the spectral power of these input signals is mainly concentrated below 0.1 Hz (i.e. the PDM characteristics below 0.1 Hz will be more influential). To examine this viewpoint, we compute the root-mean-square (RMS) contributions of each PDM for the respective input (equal to their SD value because the time-series data are demeaned) and compare them using a paired t-test. The results are shown in Table 4 and Table 5 and show that the significant differences are found in the CFV contributions of the 1st PDM of the ABP-to-CFV relation and the 3rd PDM of the CO2-to-CFV relation. The implications of these results are discussed below.

**TABLE 4:** Mean (SD) of Root-Mean-Square contributions of PDMs of the ABP-to-CFV relation before and after cholinergic blockade. Δ denotes the mean difference before minus after blockade.

ABP	PDM #1		PDM #2		PDM #3		PDM #4	
	Before	After	Before	After	Before	After	Before	After
µ (ϭ)	2.0190 (1.0214)	0.9582 (0.5337)	0.6539 (0.6763)	0.2911 (0.2009)	0.2250 (0.1854)	0.3270 (0.1529)	0.3468 (0.2424)	0.3506 (0.442)
Δ	1.0608 (0.9744)		0.3627 (0.7777)		−0.1019 (0.2309)		−0.0038 (0.5598)	
P	0.01142		0.19927		0.22197		0.98425	

**TABLE 5:** Mean (SD) of Root-Mean-Square contributions of PDMs of the CO2-to-CFV relation before and after cholinergic blockade. Δ denotes the mean difference before minus after blockade.

CO2	PDM #1		PDM #2		PDM #3		PDM #4	
	Before	After	Before	After	Before	After	Before	After
µ (ϭ)	0.9308 (1.0969)	0.4509 (0.3270)	0.4581 (0.3579)	0.4485 (0.3219)	0.1836 (0.1140)	0.5613 (0.2985)	0.1071 (0.1125)	0.3243 (0.3696)
Δ	0. (0.9815)		0.0096 (0.5131)		−0.3777 (0.352)		−0.2173 (0.4248)	
P	0.18054		0.95672		0.01225		0.16350	

## Discussion and conclusions

This paper presents a model-based methodology for the quantification of the effects of cholinergic blockade upon the dynamics of cerebral flow autoregulation (CFA) with respect to spontaneous changes of arterial blood pressure (ABP) and blood CO2 tension (CO2) (represented by the proxy of end-tidal CO2) at rest in healthy adults. The employed modeling methodology uses the canonical convolutional form (valid for all linear time-invariant systems) with two inputs (ABP and CO2) and one output of cerebral blood flow velocity (CFV) measured at one of the middle cerebral arteries *via* transcranial Doppler, and seeks the estimation of the two kernels in the model *via* Laguerre expansions (for robust estimation) using 5 min of recorded time-series data before and after cholinergic blockade in 9 subjects.

The effects of the cholinergic blockade upon the CFA dynamics are quantified by the kernel changes (or, equivalently, by changes in their frequency-domain representations of Transfer or Gain Functions) in the two causal pathways: ABP-to-CFV and CO2-to-CFV. These effects are further examined *via* Principal Dynamic Mode (PDM) analysis that decomposes the kernels of the cohort in terms of their main dynamical components. Statistical evaluation of the changes due to blockade takes place through paired t-tests.

The main findings of this study are:

1) The kernel analysis of the CFA dynamics using the linear two-input (ABP & CO2) and one-output (CFV) model, before and after cholinergic blockade, indicates significant changes in the waveforms of both kernels based on a paired t-test (see Figure 2).
2) The average Gain Function changes (due to cholinergic blockade) indicate a downshift of the resonant peaks and Gain reduction for both causal pathways: ABP-to-CFV and CO2-to-CFV (see bottom panels of Figure 2). For the ABP-to-CFV pathway, this downshift is consistent with the notion that the cholinergic mechanism has a high-pass frequency-response characteristic with resonant peak around 0.2 Hz. The average Gain reduction over most frequencies (after blockade) indicates an average increase of cerebrovascular impedance (the inverse of the ABP-to-CFV Gain Function) and an average decrease of dynamic CO2 vasomotor reactivity (related to the area under the CO2-to-CFV Gain Function).
3) The average kernel differences (before minus after cholinergic blockade) indicate distinct resonant peaks in their frequency-domain representations at 0.22 Hz for the ABP-to-CFV relation and at 0.06 Hz for the CO2-to-CFV relation (see Figure 3). The former is consistent with the finding #2 above. We note that the presence of possible nonlinearities may affect somewhat the precise location of the *apparent* peak in the DFT magnitude of the kernel difference, since the nonlinear terms are expected to influence the Gain Function of the linear approximation (i.e. the *apparent* Gain Function) (Marmarelis, 2004).
4) The average spectra of the ABP and CO2 inputs (see Figure 5) and of the CFV output (see Figure 6, blue line) indicate that cholinergic blockade has marked impact around the ABP and CFV spectral peaks at 0.05 Hz and 0.09 Hz, as well as around the CO2 spectral peak at 0.04 Hz.
5) The comparison of the average Gain Function estimates obtained *via* the Laguerre expansion technique *versus* the conventional Transfer Function method (Claassen et al., 2016) reveals marked differences in these estimates for the reasons discussed further below.
6) PDM analysis of these kernel estimates reveals significant changes (due to cholinergic blockade) in the 1st and 3rd PDM expansion coefficients for the ABP-to-CFV relation and in the 3rd PDM expansion coefficient for the CO2-to-CFV relation (see Table 2,Table 3). The 1^st^ PDM of ABP-to-CFV resembles the kernel difference of Figure 3.
7) When the PDM contributions to the model-predicted CFV output (i.e. the convolution of each PDM with the respective input) are used to assess the impact of cholinergic blockade, then statistically significant changes (p < 0.05) emerge only in the 1^st^ PDM contribution for the ABP-to-CFV relation and in the 3^rd^ PDM contribution for the CO2-to-CFV relation (see Tables 4,Table 5).
8) In the steady-state (see Time-Average/baseline values of the variables reported in Table 1), cholinergic blockade appears to increase significantly (p < 0.05) the baseline values of ABP, CFV and HR—as expected, since it reduces the inhibitory vagal stimulation of the heart—and decreases the baseline value of CO2, but not in statistically significanty manner (p=0.069). Furthermore, the blockade appears to increase the steady-state cerebrovascular resistance on average, as attested by the ratios of the respective Time-Average values for ABP and CFV over the entire cohort (both being significantly increased (p < 0.05) after cholinergic blockade).

Some key discussion points:

### The dynamic effects of the cholinergic mechanism on the regulation of cerebral flow

The effect of cholinergic blockade on dynamic autoregulation of cerebral flow is depicted in Figure 2 (in both time and frequency domains). In the frequency domain, the peak of the Gain Function is downshifted after blockade for both input pathways, as mentioned earlier. This downshift is consistent with the results of the subtractive analysis (before minus after blockade) shown in Figure 3, which suggest a possible resonant peak around 0.22 Hz for ABP-to-CFV and 0.06 Hz for CO2-to-CFV. In the context of conventional frequency-domain analysis of dynamic cerebral autoregulation (dCA) (Claassen et al., 2016), this finding suggests that the ratio of the average Gains over the range of low frequencies (0.03–0.07 Hz) to high-frequencies (0.07–0.15 Hz) will increase after cholinergic blockade—indicating deviation from normal dCA. We also see in Figure 5 that the blockade causes substantial reduction in the variability of ABP and CO2 (reduction of the integrated spectra) and particularly affects the spectral peaks around 0.04 Hz and 0.09 Hz for ABP, as well as around 0.04 Hz for CO2. Thus, cholinergic blockade causes substantial reduction in the intra-subject variability of ABP and CO2, as well as the variability of CFV that is driven by them. The reduction (after blockade) of the Gain Function values across most frequencies (see bottom panels of Figure 2) indicates a weakening of the effect of variations of ABP and CO2 upon CFV. Furthermore, Table 1 shows that the average (baseline) values for ABP and CFV are increasing significantly after blockade (along with the average heart-rate), as well as their inter-subject variability (as opposed to intra-subject variability) for both ABP and CO2. These observations suggest that the cholinergic mechanism enhances the effect of ABP and CO2 variations upon CVF and promotes intra-subject variability in cerebral perfusion. The latter may be a desirable attribute of normal physiology. These findings further suggest that strong spontaneous oscillations in the frequency ranges of 0.02–0.05 Hz and 0.08–0.10 Hz for ABP, and 0.02–0.04 Hz for CO2, are indicative of an intact cholinergic mechanism.

### Translational relevance of these findings and their potential clinical impact

Although this is a preliminary study (with a small cohort), its main findings suggest realistic potential of using Gain Function estimates as the quantitative means of assessing the level of cholinergic activity in cerebral flow autoregulation by computing the relative “distance” of an individual estimate from the two average templates shown in Figure 2. This can be used for diagnostic purposes and, in the event of cholinergic impairment, for the purpose of monitoring the progression of the impairment or the effects of possible interventions. Another translational prospect is the use of the spectral peak of the Mayer wave spontaneous oscillations of ABP or CFV (in the frequency range of 0.08–0.10 Hz), as a measure of the level of cholinergic activity (see blue lines in the left panels of Figures 5, 6). In the case of the CFV spectrum, the level of model-prediction residuals (computed through the proposed dynamic modeling approach) can be used for calibrating the magnitude of the Mayer wave estimate in order to mitigate errors due to the large physiological variability of these measurements. We note that the normalized mean-square error of the model prediction using estimates from the conventional approach usually exceeds 100%, thus making them unsuitable for this purpose.

### Subtractive analysis of the cholinergic mechanism (before minus after blockade)

As indicated in the bottom-left panel of Figure 3, the change caused by cholinergic blockade in the average Gain Function of the ABP-to-CFV kernel difference exhibits a resonant peak at 0.22 Hz, which is consistent with the view of cholinergic (vagal) drive of breathing. However, it is also known that the myogenic mechanism in the perivascular smooth muscle exhibits similarly a resonant peak in the same neighborhood of frequencies and plays a critical role in cerebral autoregulation (Aaslid et al., 1989; Paulson et al., 1990; Tiecks et al., 1995; Panerai, 1998; Zhang et al., 1998; van Beek et al., 2008). This serves as a reminder that physiological mechanisms may not be distinguishable in terms of the frequency range in which they are mostly active.

As indicated in the bottom-right panel of Figure 3, the change caused by cholinergic blockade in the average Gain Function of the CO2-to-CFV kernel difference exhibits a resonant peak around 0.06 Hz. This finding invites the search for physiological mechanisms that are active/influential around this frequency with regard to the chemoreflex and CO2 vasomotor reactivity. There is not much reported in the literature with regard to the dynamics of the CO2-to-CFV relation to allow some plausible interpretation of this finding. Nonetheless, this finding suggests that the cholinergic branch of the chemoreflex may exhibit such a frequency-response characteristic, which may be physiologically important because the CO2 spectrum of spontaneous breathing exhibits considerable power in the vicinity of this frequency (see Figure 5). We also note that the average Gain Function of the ABP-to-CFV relation exhibits a minimum around 0.06 Hz.

### The cholinergic mechanism affects more than a single PDM in the ABP-to-CFV relation

This finding suggests that we may not expect each PDM to correspond to a distinct physiological mechanism. However, the results of the PDM analysis of the CO2-to-CFV relation indicate that only the 3^rd^ PDM (see Table 3) is affected by cholinergic blockade in that input-output pathway. Furthernore, it is seen in the right panel of Figure 8 (green line) that this 3^rd^ PDM exhibits a resonant peak around 0.05 Hz, which is close to the resonant peak seen in the DFT magnitude of the CO2-to-CFV kernel difference in Figure 3. Although the interpretation of the derived PDMs is a matter of considerable complexity and will require intensive future studies, it is reasonable to posit that proper experimentation blocking a specific mechanism (as was done in this study with the cholinergic mechanism) may reveal valuable knowledge that can be used for this purpose.

### Comparison of average Gain Function estimates obtained *via* the Laguerre expansion technique *versus* the conventional transfer function method

Comparison of the average Gain Function estimates obtained *via* the two approaches (Laguerre expansion vs. conventional Transfer Function analysis (Claassen et al., 2016)) reveals marked differences (see the bottom panels of Figures 2 *versus* Figure 4). In order to explain the marked variability of the average conventional Gain Function estimates, we note that these estimates may be numerically unstable at higher frequencies where the input signals have negligible power (due to division by very small numbers). For this reason, they are plotted in Figure 4 only over the limited frequency range 0–0.25 Hz. Even over this limited range, some numerical instabilities are indicated by the large SD values (dotted lines) at certain frequencies. Furthermore, it is noted that the conventional approach does not take into account the contemporaneous cross-effects of changes in the other input—whereas the Laguerre expansion technique takes into account these contemporaneous cross-effects though the use of two inputs in the model (see Eq. 1). This may account for much of the notable differences in the average Gain Function estimates (and in their SD bounds) using these two approaches. The larger SD bounds suggest that the conventional estimates have larger inter-subject variability—and they also have larger intra-subject estimation variance, as attested by the fact that the normalized mean-square error of the conventional model prediction usually exceeds 100%. We also note marked differences between the two types of estimates in terms of magnitude scale, especially for frequencies >0.05 Hz, which may also be due to the high inter-subject variability of the conventional estimates.

It should be noted that there have been additional approaches in previous studies of cerebral autoregulation (the ABP-to-CFV dynamic relation, without accounting for contemporaneous CO2 variations) that were thoughtfully compared in (Panerai et al., 1999). In this latter paper, the first application of Laguerre expansions to this problem was also reported. The use of multivariate autoregressive methods was recently presented in connection with the effects of respiration to this system (Porta et al., 2022).

### Limitations of this study

The employed dynamic model was linear, although the physiological system under study is thought to exhibit nonlinearities. This is a limitation of the present study, which can be overcome in the future because the presented methodology is extendable to nonlinear dynamic systems -- provided longer time-series data can be collected. Another limitation is the inclusion in the model of only two “inputs” (ABP and CO2) influencing the CFV “output”, although it is known that the latter is influenced by more physiological variables (e.g. oxygen and respiration). Again, this limitation can be overcome because the presented methodology is extendable to a multi-input context -- provided the requisite time-series data from additional “inputs” can be collected. Finally, the size of the cohort in this study was small. Thus, the validation of the presented results requires a larger cohort size with the proper diversity of age and gender.

## Conclusion

The main conclusions of this study are:

1) Cholinergic blockade causes significant changes in the dynamics (kernel waveforms or Transfer Functions) of cerebral flow autoregulation in response to spontaneous variations of arterial blood pressure (ABP) and blood CO2 tension (CO2).
2) It also causes notable reduction in the intra-subject spontaneous variability of ABP, CO2 and cerebral flow velocity (CFV) at the middle cerebral arteries.
3) The Gain Function corresponding to the kernel changes (due to cholinergic blockade) exhibit a downshift of resonant peaks for both ABP-to-CFV and CO2-to-CFV pathways and result in weakening of the effect of ABP and CO2 variations upon variations of CFV.

## Data Availability

The raw data supporting the conclusion of this article will be made available by the authors, without undue reservation.
