# Supplementary material for: Dynamic effects of cholinergic blockade upon cerebral blood flow autoregulation in healthy adults
Source: Front Physiol. 2022 Nov 2;13:1015544. doi: 10.3389/fphys.2022.1015544 (PMC9666788; doi:10.3389/fphys.2022.1015544)
Supplement: Supplementary file 1 [file Presentation1.pdf]

APPENDIX : ***Kernel-based input-output modeling methodology with Laguerre expansions.***

The employed kernel-based modeling methodology is the first-order (linear) case of a general methodology for nonlinear dynamic modeling that is applicable to all finite-memory stationary dynamic systems [19]. In the linear context of the present study, the output signal $y(n)$, denoting the CFV time-series data, is expressed in terms of the input signals $p(n)$ and $x(n)$, denoting the ABP and $CO2$ time-series data, respectively, by the general linear model form:

$$y(n) = k_0 + \Sigma_m \; k_p(m)\, p(n-m) + \Sigma_m \; k_x(m)\, x(n-m) + \varepsilon(n) \qquad (A1)$$

where the summation takes place from $m=0$ to $m=M$, with M denoting the "effective memory" of the system (i.e. the maximum lag for which the kernel retains significant values), $\varepsilon(n)$ denotes the model-prediction errors (residuals), and k_0 is a constant (to be estimated). We seek to estimte the kernels $k_p(m)$ and $k_x(m)$ from the input-output data. To this end, we expand the kernels on bases of Laguerre orthogonal functions { L^p_j } and { L^x_j }, respectively, (with distinct parameters "alpha", determined from the data) [18-19] as:

$$k_p(m) = \Sigma_j \; c^p_j \, L^p_j(m) \qquad (A2)$$

$$k_x(m) = \Sigma_j \; c^x_j \, L^x_j(m) \qquad (A3)$$

where { c^p_j } and { c^x_j } denote the Laguerre expansion coefficients that will be estimated from the data (see below). The choice of the orthogonal Laguerre basis functions for the expansion of each kernel is due to the fact that these basis functions are products of an exponential term with specific polynomials (determined by the orthogonality requirement for this basis), so that they are suitable for approximating the kernels of physical systems that usually decline exponentially (in absolute value) after a certain finite lag value [18]. The rate at which the Laguerre functions decline (i.e. the negative exponent of the exponential term) is characteristic of each given kernel and must be determined in practice from the input-output data through a search procedure minimizing the prediction mean-square error [18-19]. This Laguerre kernel expansion transforms the input-output relation (A1) into Eq. (A4) that involves *linearly* the unknown Laguerre expansion coefficients:

$$y(n) = k_0 + \Sigma_j \, c^p_j \; v^p_j(n) \; + \; \Sigma_j \, c^x_j \; v^x_j(n) + \varepsilon(n) \qquad (A4)$$

where:

$$v^p_j(n) = \Sigma_m \; L^p_j(m)\, p(n-m) \qquad (A5)$$

$$v^x_j(n) = \Sigma_m \; L^x_j(m)\, x(n-m) \qquad (A6)$$

Since { $v^p_j(n)$ } and { $v^x_j(n)$ } can be computed from the respective input data according to Eq. (A5-6) and the Laguerre expansion coefficients enter linearly in the input-output model of

Eq. (A4), their estimation can be achieved via least-squares regression (a simple and robust numerical procedure) that minimizes the sum of the squared residuals $\{\varepsilon(n)\}$. In practice, these computational and estimation tasks take place in discrete time and, therefore, we must utilize the discrete form of the orthogonal Laguerre functions that are given by the expression [18-19]:

$$b_j(m) = \alpha^{(m-j)/2}(1-\alpha)^{1/2}\sum_{k=0}^{j}(-1)^k\binom{m}{k}\binom{j}{k}\alpha^{j-k}(1-\alpha)^k$$

where $b_j(m)$ denotes the j-th order discrete Laguerre function (i.e. $L^p{}_j(m)$ or $L^x{}_j(m)$) in Eqs. A5-6, computed for the respective discrete Laguerre parameter α that takes values from 0 to 1. Note that the continuous-time Laguerre parameter alpha is: $\alpha^* = -ln(\alpha)/Dt$, where Dt is the sampling interval [18-19]. The form of the first 4 discrete Laguerre functions is illustrated in Figure A1.

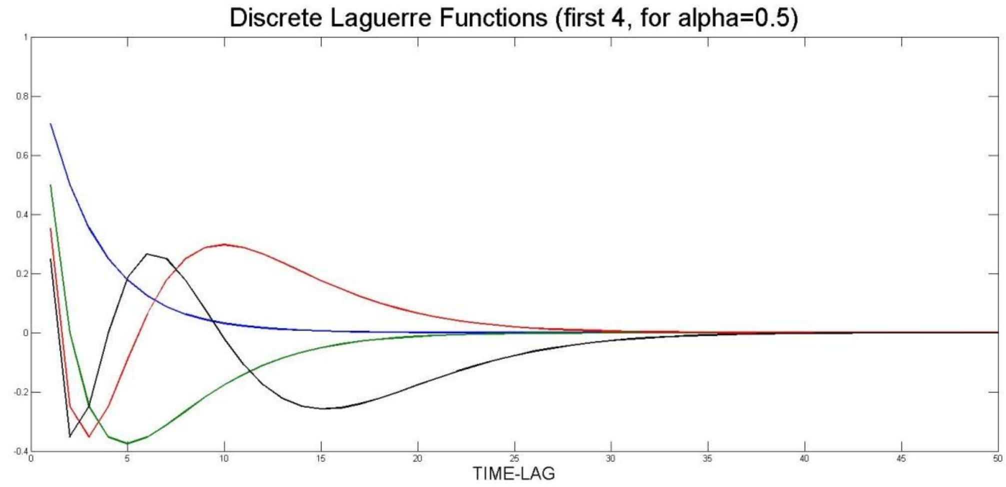

***Figure A1**: Illustration of the form of the first 4 discrete Laguerre functions for α=0.5 (in Matlab, zero lag corresponds to time-index 1). The number of zero-crossings equals the order of each discrete Laguerre function [18, 19].*

Key parameters in the application of the Laguerre expansion technique are the number of employed discrete Laguerre basis functions, as well as the discrete Laguerre parameter "alpha" that defines the relaxation dynamics of these basis functions for each input-output pair (characteristic of each input-output dynamic relation). These parameters are selected on the basis of a search procedure that seeks to minimize the Bayesian Information Criterion that takes into account the normalized mean-square error (NMSE) of the model prediction and the number of free parameters in the model. In this study, the appropriate number of Laguerre functions was found to be 4 for each input, and the Laguerre parameters alpha: 0.5 and 0.8 for ABP and CO_2, respectively. Following estimation of the Laguerre expansion coefficients, we can construct the kernel estimates using Eqs. (A2-3) and compute the model prediction for *any* given input using Eq. (A1) or (A4).

An important concept in our analysis pertains to the Principal Dynamic Modes (PDMs) of each

input-output relation that result from Singular Value Decomposition (SVD) of a rectangular matrix composed of all respective kernel estimates of a cohort (as column vectors). The PDMs are the singular vectors of the SVD result and their relative importance (in terms of relative contribution to the system kernel estimates) is quantified by the respective singular values. Thus, the PDMs represent an orthogonal basis for the expansion of each kernel and they are ranked according to their relative contribution to it, when all available kernel estimates of the cohort are taken into account. For example, the 1st PDM is the one that is found most often as a component in the cohort kernels, followed by the 2nd PDM as the next most often found as a component in the cohort kernels, and so on in an orthogonal hierarchy of steepest convergence (i.e. if one is to truncate the kernel expansion to a subspace, then the PDMs offer the optimum subspace of any given dimension).
